# Testing Low-Density Polyethylene Membranes for Lithium Isotope Electromigration System

**DOI:** 10.3390/ma18112519

**Published:** 2025-05-27

**Authors:** Andreea Maria Iordache, Ramona Zgavarogea, Ana Maria Nasture, Erdin Feizula, Roxana Elena Ionete, Rui Santos, Constantin Nechita

**Affiliations:** 1 ICSI Analytics Department, National Research and Development Institute for Cryogenics and Isotopic Technologies-ICSI, 4 Uzinei Street, 240050 Râmnicu Vâlcea, Romania; andreea.iordache@icsi.ro (A.M.I.); ramona.zgavarogea@icsi.ro (R.Z.); roxana.ionete@icsi.ro (R.E.I.); 2 ICSI Energy Department, National Research and Development Institute for Cryogenics and Isotopic Technologies-ICSI, 4 Uzinei Street, 240050 Râmnicu Vâlcea, Romania; ana.nasture@icsi.ro; 3 Biotechnologies Department, Faculty of Chemical Engineering and Biotechnologies, 313 Splaiul Independentei, 060042 Bucharest, Romania; erdin.feizula@analytik-jena.com; 4 Analytik Jena GmbH at CIQUP, Faculdade de Ciências da Universidade do Porto, Rua Campo Alegre 687, 4169007 Porto, Portugal; rui.santos@analytik-jena.com; 5 Department of Biometry, National Research Institute in Forestry Marin Dracea-INCDS, 077190 Voluntari, Romania

**Keywords:** electromigration process, Li-ion mobility, separation and purification of lithium, ^6^Li/^7^Li, ^6^Li enrichment

## Abstract

The growing energy demand has emphasized the importance of developing nuclear technologies and high-purity lithium isotopes (^6^Li and ^7^Li) as raw materials. This study investigates how voltage and migration time affect two types of low-density polyethylene membranes—one impregnated with ionic liquids and the other non-impregnated—for lithium isotope separation via electromigration from a lithium-loaded organic phase to an aqueous solution. We developed a laboratory-made setup for high-precision lithium isotope measurements (2RSD = ±0.30‰) of natural carbonate samples (LSVEC) and an optimized protocol for isotope ratio measurements using quadrupole ICP-MS with the sample-standard bracketing method (SSB). The results document that both impregnated and non-impregnated membranes can achieve promising ^6^Li enrichment under different environmental conditions, including ionic liquids and organic solutions in the cathode chamber. Lithium-ion mobility is influenced by voltage in an environment assisted by 0.1 mol/L tetrabutylammonium perchlorate and increases quasi-linearly from 5 to 15 V. Between 20 and 25 h, the lithium-ion concentration had the maximum value, after which the trend declined. In the BayesGLM model, we incorporated all data and systematically eliminated those with a low enrichment factor, either individually or in groups. Our findings indicated that the model was not significantly affected by the exclusion of measurements with low α. This suggests that voltage and migration time are crucial, and achieving a better enrichment factor depends on applying the optimal ratio of ionic liquids, crown ethers, and organic solvents. Ionic liquids used for impregnation sustain enrichment in the first hours, particularly for ^7^Li; however, after 25 h, ^6^Li demonstrated a higher enrichment capacity. The maximum single-stage separation factor for ^6^Li/^7^Li was achieved at 24 and 48 h for an impregnated membrane M2 (α = 1.021/1.029) and a non-impregnated membrane M5 (α = 1.031/1.038).

## 1. Introduction

The increasing demand for eco-friendly energy production and alternative sources that can replace fossil fuels has spurred investigations into various technologies. Current literature indicates that the global energy transition focuses on shifting energy generation from fossil fuels to nuclear fission. In this context, the separation and enrichment of lithium isotopes (^6^Li and ^7^Li) are being explored as sustainable technologies for nuclear energy production. Lithium isotope separation using the electromigration technique leverages the natural differences in migration rates of ^6^Li and ^7^Li isotopes [1]. Despite numerous efforts to develop environmentally friendly methodologies for the separation of lithium isotopes in industrial processes, the outcomes to date have been unsatisfactory [2]. Several factors contribute to these limitations, including (i) the high costs of measurement equipment, (ii) significant reagent costs associated with multiple tests, (iii) the time-consuming nature of experiments, often yielding no results, and (iv) the necessity for interdisciplinary collaboration among scientists [3,4]. Technological processes, including elevated energy consumption, harmful emissions, discharge of alkaline wastewater, substantial solvent costs, and chemical emissions, impose additional limitations [5,6,7].

Electromigration is an effective technique for lithium-isotope separation [8]. Previous experimental attempts have shown that electromigration can successfully separate lithium by regulating the migration of lithium ions (Li^+^) at the interfaces of the anode solution, organic solution, and cathode solution [9]. It was noted that attaching lithium to membranes poses a technological challenge due to its high reactivity and limited interaction with conventional membrane materials. Also, it is essential to expand material options to increase ionic selectivity and improve system performance. This requires shifting from traditional polymers, which have selectivity and stability limitations, to advanced nanoscale materials. Expanding material options for hybrid systems, nanostructures, and customized materials is essential for improving ionic selectivity and overcoming lithium permselectivity challenges [2,10]. Experiments on ionic liquid ratios have established a better understanding of lithium-isotope separation. Observations indicate that ionic liquid–crown ether systems yield better outcomes in multistage electromigration separation [8,11,12]. The migration of Li^+^ in organic solutions is complex; however, increasing the lithium-ion concentration induces differences at both ends of the organic solution (near the LiNT*f*_2_ solution and the NH_4_Cl solution). Results show a higher average concentration of Li^+^ in the organic phase as the ionic liquid concentration increases. Moreover, varying the voltage can improve the cathode solution ability (NH4Cl) to enrich ^6^Li. Also, a transition from ^7^Li enrichment at 0 V to ^6^Li at 16 V was observed, mainly because of a decrease in the bonding strength between Li^+^ and crown ethers [1]. A limitation identified in the literature concerning lithium-isotope separation via electromigration is the absence of studies that utilize statistical modeling functions. These functions could reduce the number of experiments required, given the high costs of chemicals, to predict critical factors and assess the significance of other environmental variables in achieving optimal separation efficiency.

We utilized Bayesian functions for generalized linear modeling to statistically evaluate Δ^6^Li enrichment based on two variables: voltage and migration time. This analysis aims to demonstrate (1) the optimal values for each variable and (2) assess how other factors, such as ionic liquids, organic solutions, crown ethers, and membrane characteristics, induce significant changes in the separation process reported to voltage and migration time. We hypothesize that δ^6^Li enrichment will be significantly influenced by these controlled environmental factors. This study used seven low-density polyethylene membranes (impregnated with ionic liquids before electromigration and non-impregnated) under various experimental conditions, including ionic liquid concentrations, organic solutions, and crown ethers. Additionally, it presents a novel Q-ICP-MS protocol for measuring lithium isotope ratios, achieving an impressive long-term precision of 0.30‰ and demonstrating matrix tolerance. The electromigration separation of lithium isotopes was tested in a system utilizing two crown ethers (4-nitrobenzo-15-crown-5-ether and 15-crown-5).

## 2. Materials and Methods

### 2.1. Chemical Reagents

All chemicals used in the electromigration experiments were of the highest purity. Lithium carbonate (Li_2_CO_3_, ≥98%, IAEA, LSVEC, Vienna, Austria), anisol (99.8%), and ammonium chloride (NH_4_Cl, 99.999%) were used (Sigma-Aldrich, Munich, Germany). The 4-nitrobenzo-15-crown-5-ether (4-NO_2_-B15C5; 99%) was obtained from Tokyo Chemical Industry Co. Ltd. (Tokyo, Japan), and 15-crown-5 (15C5; 98%) was sourced from Sigma Aldrich, Munich, Germany. The 1-butyl-3-methylimidazolium bis (trifluoromethylsulfonyl)imide (Sigma-Aldrich, Buchs, Switzerland), bis(trifluoromethane)sulfonimide lithium salt ([BMIm][NT*f*_2_], 99%), Anisol (99.8%), ammonium chloride (NH_4_Cl, 99.99%) and lithium salt bis(trifluoromethane)sulfonimide (LiNT*f*_2_; 99%) were obtained from Sigma-Aldrich, Buchs, Switzerland. Other reagents included tetrabutylammonium perchlorate (Fluka Analytical, London, UK), perchloric acid (70–72%, Sigma-Aldrich, Munich, Germany), and nitric acid (Suprapur 65%, Sigma-Aldrich, Buchs, Switzerland). Tetrabutylammonium perchlorate (TBAP) and CH_3_CN (acetonitrile) enhanced the electrochemical processes and provided high conductivity without interfering with the electrochemical reactions.

### 2.2. Experimental Design in a System with Crown Ethers

**Figure 1:**
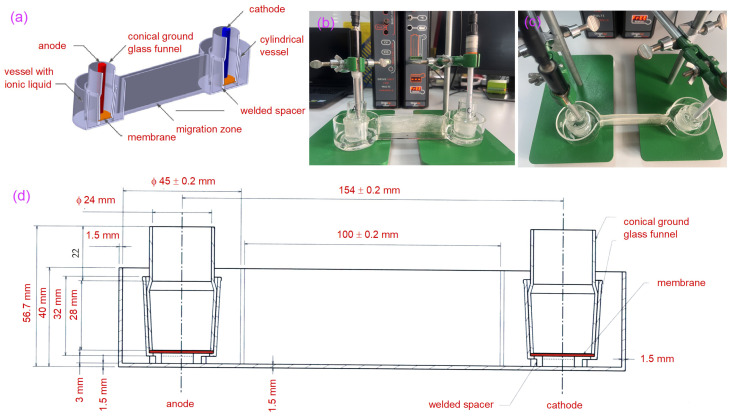
Illustration of experimental electromigration facility (**a**) transversal cross-section through experimental setup; (**b**) the experimental setup in the laboratory facility lateral view; (**c**) shows the top view of the electrode assembly fixed with support in the borosilicate glass electromigration facility system; (**d**) geometry of the experimental setup developed utilizing Computer-Aided Design (CAD) software.

Considering the expenses associated with chemicals and the need to regulate the precise concentration of each reagent and the extensive number of repetitive tests required, we designed a custom electromigration tank for the experiment with the following specifications: L = 22.5 cm (length), anode and cathode chamber diameter (ø) = 48 mm (Figure 1c,d). The geometry of the experimental setup was developed utilizing Computer-Aided Design (CAD) software (version 0.2.1.2), and after theoretical validation, it was transposed to the experimental laboratory-scale system. The custom laboratory-made tank was fabricated from borosilicate glass, chosen for its non-corrosive properties to prevent reactions with the chemical compounds used in the experiments. The materials used need to serve as the visualization and identification of the processes and reactions in real time.

Figure 1c illustrates the components of the experimental electromigration tank, which include cylindrical vessels that communicate and have an inner diameter of 48 mm and a volume of about 69 cm^3^. These vessels serve as containers for the ionic aqueous solution. The wall facilitating communication between the vessels measures roughly 100 mm in length, with an ionic liquid communication section of 3 mm, ensuring a controlled migration area and safe containment of the liquid. Inside each communicating cylindrical vessel is a conical ground glass funnel (NS 29/32), which forms the anode and cathode chambers. Conical vessels are anchored to the base of the cylindrical vessels using three 3 mm high spacers, creating a space that facilitates the free circulation of the ionic aqueous solution around the conical vessel. The catalytic exchange membrane (polymeric structure for selective ion transport and electrochemical catalysis reactions) situated in each of the conical vessels is affixed to the inner collar of the NS 29/39. It is secured in place by the alternate NS 29/32 cylinder. A comparable electromigration tank with varying specifications has been documented in the literature (L = 100 mm, r = 24 mm; h = 30 mm) [12]. Furthermore, another study incorporated a polypropylene Li battery membrane in a liquid–crown ether system, illustrating the benefit of minimizing crown ether solution loss, as it was not directly exposed to the aqueous solution [13].

### 2.3. Ionic-Liquid-LDPEs

We conducted lithium-isotope separation via electromigration utilizing a crown ether system and seven low-density polyethylene membranes (LDPE, Carl Roth, Karlsruhe, Germany; 46 μm thick, 0.4 μm pore diameter) as the raw material. Three membranes (M1, M5, and M7) were non-impregnated before the electromigration process, while the other four (M2, M3, M4, and M6) were impregnated (Table 1). The organic phase was prepared by mixing [BMIm][NT*f*_2_] and anisole in a 7:3 (*v*/*v*) ratio.

For the non-impregnated membranes, we used 1 mol/L solution of Li[NT*f*_2_] in the organic phase, which was added to the anode chamber, and a 0.005 mol/L NH_4_Cl aqueous solution in the cathode chamber. A 0.5 mol/L or 1 mol/L Li[NT*f*_2_] solution in 0.1 mol/L TBAP in acetonitrile (ACN) was used. The addition of two different crown ethers improved the migration ratio of Li^+^ from the anode solution to the organic solution, enhancing the enrichment of ^6^Li. The two crown ethers, 4-NO_2_-B15C5 and 15C5, were dissolved in the organic solution to achieve a final concentration of 0.2 mol/L, and this mixture was used for treating all membranes. We evaluated electromigration with M1 at 1 mol/L Li[NT*f*_2_] in the organic solution for the anode chamber and a 0.005 mol/L NH_4_Cl aqueous solution for the cathode chamber. For non-impregnated membrane M5*,* the anode chamber solution was 0.5 mol/L Li[NT*f*_2_] in 0.1 mol/L TBAP in CH_3_CN, while for M7, it was 1 mol/L Li[NT*f*_2_] in 1 mol/L TBAP in CH_3_CN. For M5 and M7*, *15C5 was dissolved in the organic solution to a final concentration of 0.2 mol/L.

The impregnated membranes were soaked for 2.5 h in a 1 mol/L Li[NT*f*_2_] organic solution. For M6, the membrane was activated for 2 h and then soaked for an additional 24 h in a 0.5 mol/L Li[NT*f*_2_] solution. The organic solution for the electromigration process in the anode chamber consisted of 1 mol/L Li[NT*f*_2_] or 0.5 mol/L Li[NT*f*_2_] in 0.1 mol/L TBAP in CH_3_CN. Then, 4-NO_2_-B15C5 was dissolved in the organic phase to prepare a 0.2 mol/L crown ether solution. We soaked the initial impregnated membranes (M2, M3, M4, and M6) in a 1 mol/L Li[NT*f*_2_] organic solution for 2.5 h. Membrane M6 was activated with H_2_SO_4_ for 2 h in 0.5 mol/L Li[NT*f*_2_] and then soaked for 24 h in a 0.5 mol/L Li [NT*f*_2_] solution in 0.1 mol/L TBAP in CH_3_CN. The anode chamber was filled with organic solution (0.5 mol/L Li[NT*f*_2_] in 0.1 mol/L TBAP in CH_3_CN). For the impregnated membranes M2, M3, and M4, 1 mol/L Li[NT*f*_2_] in the organic solution (7:3 *v*/*v*) was used for the anode chamber, with 0.5 mol/L Li[NT*f*_2_] in 0.1 mol/L TBAP in CH_3_CN for M6 and a 0.005 mol/L NH_4_Cl aqueous solution for all membranes in the cathode chamber. The crown ether 15C5 was dissolved in the organic solution to achieve a final concentration of 0.2 mol/L for M2, M5, M6, and M7. In comparison, 4-NO_2_-B15C5 was dissolved in the organic solution to a final concentration of 0.2 mol/L for M1, M3, and M4. The exact amounts of crown ether used were 1.10132 g of 15C5 for M2, M5, M6, and M7, and 0.6266 g of 4-NO_2_-B15C5 for M1, M3, and M4.

### 2.4. Experimental Procedures

An organic solution/organic solution/aqueous solution system was established for the electromigration separation of lithium isotopes. Initially, 7 mL of [BMIm][NT*f*_2_] was combined with 3 mL of anisole to create a 10 mL organic solution, which was subsequently placed in the electromigration tank. Membranes were categorized into two types before the electromigration process: non-impregnated and impregnated (Table 1). Thus, for the non-impregnated membrane M1, 1 mol/L Li(NT*f*_2_) in organic solution was added to the anode chamber, while the cathode chamber contained a 0.005 mol/L NH_4_Cl aqueous solution. In cases M5 and M7, either 0.5 mol/L or 1 mol/L of Li(NT*f*_2_) in 0.1 mol/L TBAP (tetrabutylammonium perchlorate) dissolved in CH_3_CN (acetonitrile) was used. The addition of two different crown ethers aimed to improve the migration ratio of lithium ions from the anode solution to the organic solution, thereby enhancing the enrichment of ^6^Li. A concentration of 0.2 mol/L of crown ether (4-NO_2_-B15C5/15C5) was incorporated into the organic solution. The LDPE impregnated membranes were soaked for 2.5 h in a 1 mol/L Li(NT*f*_2_) organic solution. Alternatively, they were activated for 2 h, followed by soaking for 24 h in a 0.5 mol/L solution of Li[NT*f*_2_] Bis-(trifluoromethane) sulfonimide lithium salt. The organic solution used for the electromigration process in the case of M2, M3, M4, and M6 at the anode was either 1 mol/L Li(NT*f*_2_) or 0.5 mol/L Li(NT*f*_2_) in 0.1 mol/L TBAP in CH_3_CN (Table 1).

The disposal of the ionic liquids within the electromigration tank was conducted as follows: 15 mL was placed in the anode chamber, 15 mL was allocated to the cathode chamber, and 30 mL was designated for the organic solution communication room. These chambers were separated by either commercially impregnated or non-impregnated low-density polyethylene membranes (Karlsruhe, Germany). A platinum wire electrode with a 1 mm diameter was placed in both the anode and cathode solutions. An electric field was applied using a DC power supply, Origalys potentiostat (Rillieux-la-Pape, France), at different potentials (5–15 V). Samples from the anodic and cathodic solutions were collected at 2, 6, 20, 25, and 48 h intervals. In our experiment, electrochemical studies on the transfer from ^7^Li to ^6^Li process were conducted using the chronoamperometric method.

### 2.5. Sample Digestion

Sample digestion was conducted at the Isotope Metals Laboratory within the ICSI Analytics Department at the ICSI Institute. A sample of 1 mL was collected from the anode and cathode solutions at intervals of 2, 6, 20, 25, and 48 h. The collected organic matter from both locations was subjected to dry extraction on a sand bath utilizing a mixture of concentrated nitric acid and perchloric acid (in a volume ratio of 4:1) at a temperature of 195 °C with a heating power of 70 W. The procedure was repeated, as the extracts with the organic solution exhibited small brown spots following centrifugation, indicating an incomplete dissolution of the crown ether. Following the dry digestion, the resultant solution was clear and transparent.

### 2.6. Chromatography

The chromatography system was designed in two stages to ensure high sample loading and a fixed Li elution range [13]. For the first stage, we used BioRad^TM^ Econo-Pac columns (1.5 cm ID × 12 cm H, polypropylene); for the second stage, we custom-fabricated columns (0.6 cm ID, polypropylene). All columns were filled with pre-cleaned cation-exchange resin, AG 50W–X8 resin, which has been successfully employed to separate Li, Na, and K from natural samples [14]. The dual-column system enables a high loading capacity (24.4 meq), complete recovery (~97%), and satisfactory purification (Na/Li < 1) of Li. Additionally, the dual-column setup routinely achieved a fixed elution range of Li cuts (60–105 mL 0.7 M HNO_3_). All columns were cleaned with 50 mL 6 N HCl (37% p.a.). A volume of 1 mL of 0.7 mol/L HNO_3_ was eluted through the columns and then cleaned with ~50 mL of 6N HCl. In the recovered Li solutions, we analyzed the concentrations of ^23^Na, ^39^K, ^6^Li, and ^7^Li. Aliquots of each sample were prepared in 0.7 M HNO_3_ (*v*/*v*) containing 10, 50, 100, 250, 500, and 1000 ppb of Li for quantitative Q-ICP-MS analyses, along with 10 ppb of LSVEC reference material for Li isotope ratio measurements. The 45 mL of Li fractions collected after column separation were quantitatively analyzed for ^6^Li, ^7^Li, ^23^Na, and ^39^K by Q-ICP-MS Plasma-Quant MS Elite (Analytik Jena, Jena, Germany).

### 2.7. Isotope Ratio Measurements

Lithium isotope ratios are measured by the instrument performance of ICP-Q-MS Plasma Quant Elite (Analytik Jena, Germany), a single-collector quadrupole. The instrumental settings for Li isotope ratio determination and the column specifications used for the isolation of lithium from sodium and other elements are presented in Table 2. This research employed a measurement protocol for lithium isotope ratios using the sample-standard bracketing method (SSB). Our optimized instrumental setup demonstrated high-precision lithium isotope measurements with a 2RSD of ± 0.30 ‰ for natural carbonate samples (LSVEC). We used a platinum skimmer cone to minimize carry-over effects and blanks. This section provides detailed information on achieving routine high precision and accuracy in lithium isotopic analysis using a Q-ICP-MS by optimizing various parameters. The optimal conditions for lithium isotope analysis were as follows: RF power of 0.90 kW; plasma gas flow rate of 9 L/min; auxiliary gas flow rate of 1.50 L/min; nebulizer gas flow rate of 1.00 L/min; Ar gas flow of 10 mL/min; and analysis scan mode set to peak hopping. Data acquisition was performed using 1 point and 999 scans per peak, with six replicates, which resulted in satisfactory resolution measurements. Each measurement included 10,000 µs and 130,000 µs of dwell time for ^7^Li and ^6^Li, respectively, with six replicates. Instrumental stability is critical for obtaining accurate isotopic ratios using the SSB method. The balanced bracket sequence for the analytical routine was as follows: blank, LSVEC without matrix, LSVEC with matrix, LSVEC, LSVEC without matrix, sample anode, blank bracket sequence, LSVEC, LSVEC without matrix, LSVEC with matrix, sample cathode, blank, and LSVEC.

### 2.8. Analytical Methods

The lithium isotope ratio (Δ^6^Li) was calculated by standardizing the tested sample against the LSVEC standard, which is a lithium carbonate (Li_2_CO_3_) manufactured by the National Institute of Standards and Technology [15]. The organic solution for the ^6^Li/^7^Li standard has an abundance ratio of 0.08215 ± 0.00023 (IAEA-LSVEC Lithium Carbonate, Vienna, Austria), with uncertainties expanded using a coverage factor of two. The lithium isotopic composition is expressed as Δ^6^Li, representing the per mil deviation from the NIST LSVEC standard, as defined in Equation (1).

(1) Δ6Li·%, LSVEC=L6i/L7isampleL6i/L7iSVEC−1×1000

where the *(*^6^Li/^7^Li)_sample_ represents the lithium isotope abundance ratio in samples extracted during the experiment, while *(*^6^Li/^7^Li)_standard L-SVEC_ denotes the lithium isotope abundance ratio in the L-SVEC standard. The resulting Δ^6^Li reflects the lithium isotopic values from the experiment. The separation coefficient, α, was used to indicate the lithium isotope separation effect during the electromigration process and was calculated using Equation (2).

(2) L6i sep coef αm=Li6/Li7xLi6/Li7y

where (^6^Li/^7^Li) represents the lithium isotope abundance ratio, with the subscripts *x* denoting the cathode phase and *y* denoting the anode phase in the two liquid-separation processes.

### 2.9. Statistical Analysis

The summary statistics, including the mean (±SD), were used to describe the results. We applied a generalized linear regression (BayesGLM) to predict the unknown parameters of the model using the Bayesian technique [16,17]. BayesGLMs provide an accurate description of inferences and relationships between dependent variables and one or more independent variables, especially when the response variables do not follow a normal distribution [18]. The GLMs include a link function, which helps model non-linear relationships within a linear framework, offering high flexibility and versatility. The model estimation and fitting were performed using maximum likelihood estimation (MLE). To evaluate and select the best model, we used the following parameters: goodness of fit (deviance), residual analysis (via plots), overdispersion (Chi-squared/DF), coefficient of significance (*p*-values), and predictive performance metrics [Akaike information criterion (AIC) and Bayes information criterion (BIC)]. All covariates were standardized using *z*-score, and the confidence interval was computed using the bootstrap bias-corrected and accelerated (Bca) method with 1000 replications. The dependent variable in the model was “enrichment” as the factors included time (H), voltage (Voltage), and ionic mobility (^6^Li and ^7^Li). Covariates included lithium abundance in the anode (aA) and cathode (aC). The mathematical formula for the model is provided in Equation (3). The percentage difference algorithm was used to assess differences between enrichment values obtained at 25 and 48 h, allowing us to evaluate the experiment’s efficiency over an extended period for each LDPE membrane.


BayesGLM: enrichment ~ 1 + H + Voltage + aA + aC + H:Voltage
(3)

## 3. Results and Discussion

The lithium isotope separation of the “lithium salt solution (anolyte)|crown ethers-ionic liquid organic solution|ammonium chloride aqueous solution (catholyte)” electromigration system, using 4Nitrobenzo-15-crown-5 and 15-crown-5 as transfer catalysts, was investigated. Two types of membranes were tested: initially, non-impregnated membranes (M1, M5, M7) and impregnated for 2.5 h in 1 mol/L Li[NT*f*_2_] in organic solution, followed by soaking for 24 h in 0.5 mol/L Li[NT*f*_2_] Bis-(trifluoromethane) sulfonimide lithium salt (Table 1). In the custom experimental laboratory-made electromigration system, a solution-organic and solution-aqueous medium was initially added, with a lithium salt solution concentration of 7000 mg/L in the anode chamber. The highest lithium salt concentration measured after the cessation of the experimental flow was 4622 and 3497 mg/L in the anode chamber for membranes M2 and M5. The Li^+^ migration rates from the anode to the organic phase were 0.33% and 0.50%, respectively. We are inclined to believe that TBAP 0.1 M in CH_3_CN solution will lead to better results than organic solution, since as a comparison of parameters for M2 and M5 membranes, we found no other significant differences (e.g., crown ether 15-crown-5, 0.2 mol/L, electromigration solution at the cathode of 0.005 mol HN_4_Cl and a pH before and after electromigration of 2.20/2.19 (cathode) and 6.77/6.67 (organic solution and crown ether)). Also, the membrane M3 exposed to the same 4Nitrobenzo-15-crown-5 electromigration solution had a Li+ migration rate that does not differ significantly (*p* < 0.01) from M2. In a similar study conducted on laboratory-made membranes, the TBAP (tetra-butyl ammonium perchlorate) solution in CH_3_CN was also found to increase the Li^+^ migration rates [19]. Other studies proved efficiency in achieving a higher lithium separation factor for 15-crown-5 only after crown ether customization using various polymers [20,21,22,23]. The performance of B15C5 crown ether as an extractant for lithium isotopes is attributed to its cavity size, which closely matches the ionic diameter of Li^+^ [24].

### 3.1. Driving Effects of the Migration Electric Field

**Figure 2:**
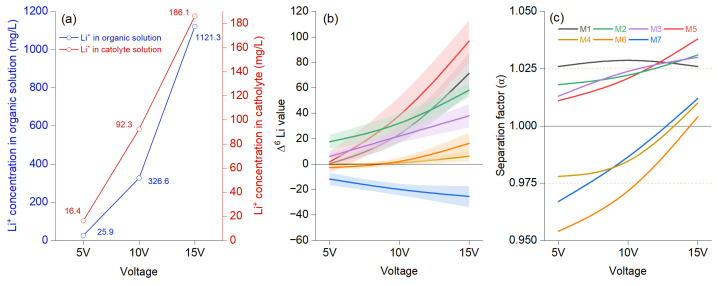
The electromigration results using voltage as reference for migration effects (**a**) show the average concentration of Li^+^ in the organic (blue color) and cathode (red color), (**b**) lithium isotope ratio at 5, 10, and 15 V voltages at the best enrichment factor under the migration time of which was achieved at 48 h for each membrane setting described in Table 1, modeled *B*-spline function to illustrate a continuous process and bands representing confidence intervals, and (**c**) the separation factor (α) represented as a modeled *B*-spline function.

The Li+ concentration in the organic solution analyzed at 48 h shows an accentuated increase in voltage after 10 V, compared to the catholyte, where the upward trend is almost linear from 5 to 15 V (Figure 2a). Comparing the initial values in the organic phase with those in the aqueous phase, ^6^Li was significantly enriched (Figure 2a), surpassing ^7^Li at the same voltage, irrespective of the membrane used. The separation factor shows an upward trend for a first group of membranes (M5, M2, M1, and M3) under the increasing voltage, and all proved values are higher than 1 from the earliest stage. The Δ^6^Li value was highest at 15 V, documenting enrichment in organic solution (Figure 2b). Nevertheless, M7 exhibited a negative trend that increases with voltage, indicating a rising Δ^7^Li value. Additionally, M4 and M6 start from a negative value and become positive at 15 V. Figure 2c shows an apparent increase only for M5 and M2, while the upward trend for M3 is less pronounced, and M1 demonstrates a downward trend. For the other three membranes, changes occurred after the voltage increased, raising the separation factor to 1 at 15 V. Increasing the voltage resulted in a higher migration ratio but a lower isotope separation factor. Previous experiments evaluating voltages between 1.5 to 20 V showed that ^6^Li enrichment positively correlated with extended durations at low voltage (1.5 V). Moreover, a linear relationship was observed between the δ^7^Li isotope value during the first 10 min and the voltage-dependent δ^6^Li value [25]. A positive correlation between voltage (ranging from 2 V to 16 V) and the separation factor was also observed [26].

The enrichment of ^6^Li in the aqueous phase compared to the organic phase can be attributed to 4-NO2-B15C5, which enhances ^6^Li transfer. Also, a positive role is attributed to ionic liquid-assisted electromigration in enhancing lithium-ion selectivity and separation efficiency [10,27]. The effect of the ionic liquid ratio in the organic solution on the membranes initially showed an increase, stabilized around 20%, and subsequently declined when the ionic liquid ratio reached 100% [1]. The same study reported that the viscosity of the organic solution was positively correlated with the proportion of ionic liquid, with a significant increase observed after the ionic liquid volume exceeded 70%. This increase in viscosity reduced the migration rate of lithium ions in the organic solution. Consequently, it is essential to carefully assess the efficiency and potential interactions between membranes, solvents, and lithium ions to maximize selectivity, mobility, and permeability [28,29]. Ionic liquid, anisole, tetrabutylammonium perchlorate, acetonitrile, and hydrophobic membranes significantly limit the migration of lithium ions into the organic solution under an electric field [30,31,32]. Crown ethers played a key role in modulating the selectivity and permeability of LDPEMs.

### 3.2. Driving Effects of the Migration Time

**Figure 3:**
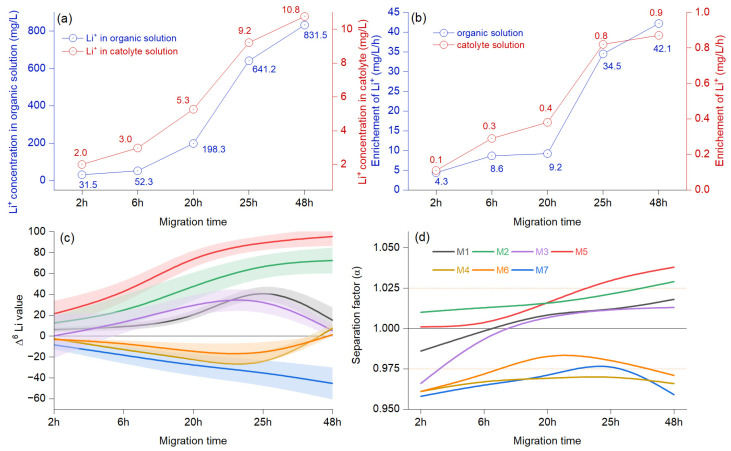
Electromigration results from assessing various migration times as reference for effects (**a**) show the average concentration of Li^+^ in the organic (blue color) and cathode (red color) at different migration time, (**b**) average Li^+^ concentration changes in relation to time, (**c**) different isotopic ratio related to migration times at 15 V, modeled *B*-spline function to illustrate a continuous process and bands representing confidence intervals, and (**d**) the separation factor (α) represented as a modeled *B*-spline function.

The concentration of Li^+^ in the organic solution rises more rapidly after 20 h, while in the catholyte solution, the sustained increase begins at 6 h, reflecting a similar overall trend (Figure 3a). The enrichment of Li^+^ documents a faster start between 2 and 6 h, followed by an almost flat trend between 6 and 20 h. The Li^+^ enrichment rate rises rapidly between 20 and 25 h, continuing in the same trend from 25 to 48 h, only in the organic solution. In contrast, the catholyte solution shows a relatively low enrichment rate after 25 h (Figure 3b). The Δ^6^Li for M2 and M5 shows a fast increase after 6 h, but between 25 and 48 h, the isotope ratio indicates a slow value (Figure 3c). The paired two-sample *t*-tests revealed no significant differences between the results obtained at 25 and 48 h (*p*> 0.05). In some cases, Δ^6^Li values decline faster (M3, M1) or show negative values for M4, M6, and M7, indicating enrichment in Δ^7^Li. A one-way ANOVA for repeated measurements revealed that Mauchly’s test for sphericity was significant (*p* < 0.0001), and the LDPE membranes M2 and M5 had an enrichment that differed significantly from the other samples tested. The results show a higher separation factor for the membranes M2 and M5. However, the difference was not statistically significant between 24 and 48 h for M2(α = 1.021/1.029) and M5 (α = 1.031/1.038) (Figure 3d). In comparison, the M1 and M3 membranes displayed reduced performance right from the start, with initial values falling below 1 and surpassing this threshold only between 6 to 20 h. Meanwhile, the M4, M6, and M7 membranes showed low separation factors, registering values in the interval of α = 0.958 and 9.86.

A difference was observed between the M2 and M5 membranes during the initial hours of the process. The M2 membrane exhibited a consistent increase in enrichment, whereas the M5 membrane remained relatively stagnant during the first six hours. This behavior can be explained by the molecular weight of the lithium ions–crown ether complex, which is higher than that of unbound lithium ions [25]. The Li^+^ migrates more quickly, especially in the early stages, as seen in the case of M2. For M5, the electromigration ratio was negligible during the first six hours but demonstrated stability after 25 h. Impregnated membranes with 1 mol/L Li[NT*f*_2_] in organic solution (M2) demonstrated higher enrichment compared to membranes impregnated with 0.5 mol/L Li[NT*f*_2_] in 0.1 mol/L TBAP in CH_3_CN (M5), only in the first hours. However, after 25 h, the ^6^Li electron migration ratio decreased substantially for M2, while M5 exhibited quasi-stable values (Figure 3). Previous tests evaluated the advantages of reducing the dissolution loss of crown ether by using simple equipment to control the migration process.

The average concentration of lithium ions in the organic solution increases with the proportion of ionic liquid, thereby enhancing wettability. The maximum single-stage separation factor for ^6^Li/^7^Li was achieved using ionic liquid and dichloroethane as the extractant. DB14C4, successfully synthesized for this purpose, demonstrated good selectivity and separation performance for lithium isotopes [33]. Our preliminary experimental results indicated that ^6^Li was enriched in both the organic and cathode solutions, achieving a maximum separation coefficient α = 1.029. This outcome arises from the coupling of diffusion and electromigration. The external precision of this method is better than 0.3‰ (2RSD) for the LSVEC standard, meeting the research requirements for separation methodologies. However, the lower mass resolution capability of ICP-MS compared to other multicollection tools may penalize the measured maximum separation factor, as highlighted in this study. The results confirm previous studies, which indicate that crown ether chelates with lithium ions in the aqueous phase, facilitating their transfer from the aqueous to the organic solution [11]. The highest separation factors for lithium isotopes, α = 1.038 and α = 1.049, were recorded in environments containing B15C5 and B12C4, respectively, based on a novel ion-pair strategy that introduced tetrachloroferrate ([FeCl_4_]^−^ as a counter-anion [34]. The straightforward stripping crossflow multistage method (SCFM) achieved extensive enrichment of the lighter ^6^Li^+^ isotope through a combination of extraction and stripping processes [10]. Some studies found that the separation coefficient achieved high values at low migration ratios, but as the migration ratio increased, the separation process became less efficient [9].

### 3.3. Modeling the Driving Effects of the Voltage and Migration Time

**Figure 4:**
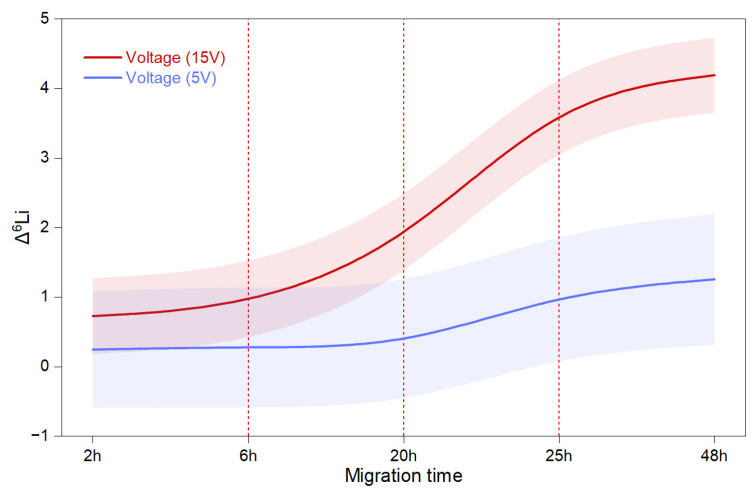
The BayesGLM model-fitting results indicate an increase in LDPE membrane enrichment performance with increasing voltage and migration time, modeled as a B-spline function to illustrate a continuous process, with bands representing confidence intervals.

We employed a flexible methodology to estimate variations in random effects estimates using a Bayesian framework, which can mitigate the drawback of small sample size. In our model, we incorporated all data and systematically eliminated those with a low enrichment factor, either individually or in groups. Our findings indicated that the model was not significantly affected by the exclusion of measurements with low α. This suggests that voltage and migration time are crucial, and achieving a better enrichment factor depends on applying the optimal ratio of ionic liquids, crown ethers, and organic solvents. The statistics of the BayesGLM model fit indicate reliability, with *R*^2^ *=* 0.47, *p* < 0.001, *Log Likelihood* = −128, *AIC* = 283, *BIC =* 312, and *Deviance* = 162. The Chi-squared/DF value of 2.8 suggests overdispersion, which can be attributed to variance exceeding the mean values (Figure 4). According to our results, the BayesGLM model, which incorporated the performance of both impregnated and non-impregnated membranes while considering environmental factors as constants, highlighted that both electric field and migration time are dominant variables influencing the separation process. The model indicated that Li^+^ remains partially associated with the crown ether environment; however, ^6^Li benefits from its faster dissociation compared to ^7^Li, leading to its preferential enrichment in the catholyte solution. Figure 4 displays the estimated marginal means, categorized by two voltage levels (5 V and 15 V) and migration times (2, 6, 20, 25, and 48 h). Our results show that high voltage has a significant effect on the enrichment factor after 6 h, with an enlarged change in the upward trend occurring between 20 and 25 h. Additionally, the model suggests that beyond 25 h, the enrichment factor continues to improve, albeit at a slower rate.

Further investigations are necessary to assess the role of crown ethers within the system and to evaluate the recyclability of the solvent, which is equally significant in constructing a reliable model for predicting factor effects on the lithium electromigration process. While peer-reviewed literature offers various approaches for the ^6^Li^+^ enrichment based on extraction separation, only a few methods provide comparable results due to variations in experimental environments. Nevertheless, it is clear that using ionic liquids as solvents can achieve better separation coefficients than diffusion and migration methods, which rank among the most effective reported techniques. Different separation methods, each based on specific theories, exhibit significant variability in performance, highlighting the need for further research to optimize lithium isotope separation strategies.

## 4. Conclusions

A “lithium salt solution (anolyte)|crown ethers-ionic liquid organic solution|ammonium chloride aqueous solution (catholyte)” electromigration system was developed for the separation of lithium isotopes. This study tested a method for lithium isotope separation, leveraging the advantages of simple ICP-MS equipment and precise control over the migration process. Voltametric measurements were conducted using supporting electrolyte solutions, such as tetrabutylammonium perchlorate (TBAP) in acetonitrile (ACN). The main conclusions are as follows: (1) both impregnated and non-impregnated LDPE membranes can achieve a promising ^6^Li separation factor, but ionic liquids’ adjustment can influence significantly the electromigration process, particularly during the initial hours of separation; (2) TBAP 0.1 M in CH_3_CN solution will lead to better results than organic solution; (3) ^7^Li was predominantly enriched in the aqueous solution during the initial hours of the experiment, whereas ^6^Li showed enrichment in the organic phase, reaching a maximum value after 25 h. Between 25 and 48 h, the enrichment is growing but at a slow rate; (4) Li^+^ concentration in catholyte solution rises almost linearly with a significant trend; and (5) the BayesGLM predictive model confirmed that among all environmental factors considered, the increasing voltage induced a linear ascending trend of Li^+^ migration in the catholyte chamber, with the highest effect observed at 25 h. Further investigations are recommended to evaluate the potential of multistage extraction processes and to optimize the balance between voltage, ionic liquids, crown ethers, and lithium isotope fractionation to enhance separation efficiency.

## Figures and Tables

**Table 1:** Experimental conditions for optimization tests for the electrochemical lithiation process (EMP = electromigration process, C[M] = molar concentration, E[V] = electric potential). The ionic liquid NT*f*_2_ (1-butyl-3-methylimidazolium bis((trifluoromethyl)sulfonyl)imide) was mixed with anisole in a 7:3 ratio *v*/*v*, and 0.2 mol/L of 15-crown-5/4 nitrobenzo-15-crown-5 ether was added to create the organic solution, which was then placed in the intermediate tank between the anode and cathode. Non-impregnated and impregnated membranes were exposed to organic solutions and different crown ethers in the electromigration process.

No. Exp.	Impregnation Before EMP	Membrane	C[M]_Li_	E [V]	Crown Ether
1	non-impregnated	M1 (LDPE-low-density polyethylene commercial membrane)	1 mol/L Li[NT*f*_2_] in organic solution	5–15	4Nitrobenzo-15-crown-5
2		M5 (LDPE-low-density polyethylene commercial membrane)	0.5 mol/L Li[NT*f*_2_] in 0.1 M TBAP/CH_3_CN	5–15	15-crown-5
3		M7 (LDPE-low-density polyethylene commercial membrane)	1 mol/L Li[NT*f*_2_] in 0.1 M TBAP/CH_3_CN	5–15	15-crown-5
4	impregnated	M2 (soaked for 2.5 h in 1 mol/L LiNT*f*_2_ in organic solution (NT*f*_2_: anisol, 7:3, *v*/*v*))	1 mol/L Li[NT*f*_2_] in organic solution	5–15	15-crown-5
5		M3 ((soaked for 2.5 h in 1 mol/L LiNT*f*_2_ in organic solution (NT*f*_2_: anisol, 7:3, *v*/*v*))	1 mol/L Li[NT*f*_2_] in organic solution	5–15	4Nitrobenzo-15-crown-5
6		M4 ((soaked for 2.5 h in 1 mol/L LiNT*f*_2_ in organic solution (NT*f*_2_: anisol, 7:3, *v*/*v*))	1 mol/L Li[NT*f*_2_] in organic solution	5–15	4Nitrobenzo-15-crown-5
7		M6 (activation with H_2_SO_4_ for 2 h, soaked for 24 h in 0.5 mol/L LiNT*f*_2_ in organic solution (NT*f*_2_: anisol, 7:3, *v*/*v*))	0.5 mol/L Li[NT*f*_2_] in 0.1 M TBAP/CH_3_CN	5–15	15-crown-5

**Table 2:** ICP-Q-MS operating parameters and column characteristics.

Parameter (ICP-MS)	Specification	Column Characteristics	Specification
Plasma gas flow	9.0 L/min	Column material	Polypropylene (Econo-Pac^®^, Bio Rad, Hercules, CA, USA)
Auxiliary gas flow	1.5 L/min	Internal diameter	1.5 mm
Sheath gas flow	0.0 L/min	Resin type	AG 50W-X8 (200–400 mesh size)
Nebulizer gas flow	1.00 L/min (MicroMistTM)	Resin volume	14 mL (wet)
Sampling depth	5.0 mm	Resin capacity	24.4 meq (1.74 meq/mL wet capacity)
Plasma RF power	0.90 kW	Resin height	80 mm
Cones	Ni cones	Flow rate	0.28 mL/min
Pump rate	10 rpm	Load sample	1 mL in 0.7 mol/L HNO_3_
Stabilization delay	30 s	Load	14–23 meq/mL
Dwell time	^6^Li—130,000 μs ^7^Li—10,000 μs	Pre-wash	6 N/L HCl (three column volume)
Scan	999	Conditioning	0.7 mol/L HNO_3_ (three column volume)
Replicates	6	Elution matrix	0.7 mol/L HNO_3_ (three column volume)
Total acquisition time	15 min	Lithium fraction	32 to 57 g
Recovery	45 mL	Lithium fraction	85–109%

## Data Availability

The original contributions presented in this study are included in the article. Further inquiries can be directed to the corresponding author. (specify the reason for the restriction).
